# Monocyte chemotactic protein-1 promotes the proliferation and invasion of osteosarcoma cells and upregulates the expression of AKT

**DOI:** 10.3892/mmr.2015.3375

**Published:** 2015-02-18

**Authors:** QUANCHI CHEN, WEI SUN, YUXIN LIAO, HUI ZENG, LIANCHENG SHAN, FEI YIN, ZHUOYING WANG, ZIFEI ZHOU, YINGQI HUA, ZHENGDONG CAI

**Affiliations:** 1 Department of Orthopedics, First People’s Hospital, Shanghai Jiaotong University, Shanghai 200080, P.R. China; 2 Department of Orthopedics, Tenth People’s Hospital of Tongji University, Shanghai 200072, P.R. China; 3 Postdoctoral Research Station of School of Life Science and Technology of Tongji University, Shanghai 200092, P.R. China

**Keywords:** osteosarcoma, monocyte chemotactic protein-1, proliferation, invasion

## Abstract

Monocyte chemotactic protein-1 (MCP-1/CCL2) is an important immune factor, which may be important in cancer progression by promoting proliferation, invasion, metastasis and the tumor microenvironment. Previous studies have demonstrated that CCL2 affects the proliferation of osteosarcoma cells via the RANKL signaling pathway. However, the underlying mechanisms remain to be elucidated. To investigate the role of CCL2 in osteosarcoma cells, MTT, spheroid forming, wound healing and transwell assays were performed to examine the proliferation and invasion abilities of the osteosarcoma cells. It was revealed that the high-grade osteosarcoma cells exhibited increased expression levels of CCL2 compared with the low-grade osteosarcoma cells (P<0.001). Furthermore, knockdown of CCL2 decreased the proliferation and invasion abilities of the osteosarcoma cells (P<0.01). These results suggested that the expression of CCL2 is high in high-grade osteosarcoma cells and promotes the proliferation and invasion of osteosarcoma cells.

## Introduction

Chemokines are a superfamily of small, secreted molecules, which exert their activity by binding to G-protein-coupled receptors. Currently, ~50 ligands and 20 receptors have been identified in humans and mice (1). Chemokines are classified into four families, CXC, CC, CX3C and C, according to the presence of four cysteine residues in a conserved region (2). It has become clear that chemokines and receptors control the proliferation and metastasis of tumor cells (3). They are considered to have a critical and complicated role in a variety of cells, including tumor cells (4,5).

Osteosarcoma is the most common type of malignant bone tumor in childhood and adolescence, and represents 15% of all primary bone tumors and 0.2% of all malignant tumors in children (6,7). The prognosis of patients with primary metastatic disease is poor (8). Tumor cells, including osteosarcoma cells, and cells in the tumor microenvironment, including fiboclasts and endothelial cells, secret monocyte chemotactic protein-1 (MCP-1/CCL2) (9). As an inflammatory medium, CCL2 is the predominant chemotactic protein, which attracts macrophages into the tumor environment from the blood. It is hypothesized that by attracting macrophages into the microenvironment and inducing these cells to release a series of factors, CCL2 promotes tumor cell generation and angiogenesis, and increases the chance of metastasis (10–13). The present study investigated the role of CCL2 in osteosarcoma cells.

## Materials and methods

### Materials

All animal experiments were performed according to the Animal Experimental Ethics Committee of Tongji University (Tongji, China) and approved by the Ethics Committee of Shanghai Tenth People’s Hospital (Shanghai, China). The rabbit antibodies used for western blotting were as follows: Anti-CCL2 rabbit polyclonal antibody (1:1,000 dilution; cat. no. sc-28879; Santa Cruz Biotechnology, Inc., Santa Cruz, CA, USA); anti-phosphorylated (p-)Akt rabbit monoclonal antibody (Ser473) (1:1,000 dilution; cat. no. 4060), anti-Akt rabbit monoclonal antibody (1:1,000 dilution; cat. no. 4685) (Cell Signaling Technology Inc, Danvers, MA, USA) and anti-β-actin rabbit polyclonal antibody (1:1,000 dilution; cat. no. A2066; Sigma-Aldrich, St. Louis, MO, USA).

### Cell culture

The LM8, Dunn, K7, K7M3 and K12 murine osteosarcoma cell lines were kindly donated by Dr Kleinerman (MD Anderson Cancer Center, Houston, TX, USA). All cell lines were cultured in high glucose Dulbecco’s modified Eagle’s medium (DMEM-h; Thermo Fisher Scientific, Waltham, MA, USA), supplemented with 10% fetal bovine serum (FBS; Thermo Fisher Scientific), 100 U/ml penicillin and 100 *μ*g/ml streptomycin (Thermo Fisher Scientific). The cultures were incubated at 37°C in a humidified atmosphere, containing 5% CO_2_.

### Knockdown of CCL2 in the LM8 cells

Short hairpin (sh)RNA constructs targeting mouse CCL2 mRNA were synthesized and termed CCL2-sh1, CCL2-sh3, CCL2-negative control (NC). The shRNA sequences used were as follows: CCL2-sh1, forward 5′-GAT CCG CAA GAT GAT CCC AAT GAG TAT TCA AGA GAT ACT CAT TGG GAT CAT CTT GCT TTT TTG-3′ and reverse 5′-AAT TCA AAA AAG CAA AGA TGA TCC CAA TGA GTA TCT CTT GAA TAC TCA TTG GGA TCA TCT TGC G-3′; CCL2-sh3, forward 5′-GAT CCG CAG GTC CCT GTC ATG CTT CTT TCA AGA GAA GAA GCA TGA CAG GGA CCT GCT TTT TTG-3′ and reverse 5′-AAT TCA AAA AAG CAG GTC CCT GTC ATG CTT CTT CTC TTG AAA GAA GCA TGA CAG GGA CCT GCG-3′ and CCL2-NC, forward 5′-GAT CCG TTC TCC GAA CGT GTC ACG TTT CAA GAG AAC GTG ACA CGT TCG GAG AAC TTT TTT ACG CGT G-3′ and reverse 3′-AAT TCA CGC GTA AAA AAG TTC TCC GAA CGT GTC ACG TTC TCT TGA AAC GTG ACA CGT TCG GAG AAC GA-3′ (Genomeditech, Shanghai, China). pGMLV-SC1 RNA interference (RNAi) vectors containing either CCL2-sh1, CCL2-sh3 or CCL2-NC were synthesized using the primers, and were transfected into HEK-293 cells, which were seeded in 96-well clusters for amplification, using Lipofectamine^®^ 2000 (Invitrogen Life Technologies, Carlsbad, CA, USA). Following transfection for 40 h at 37°C, the supernatants were collected and incubated with the LM8 cells for 24 h in the presence of polybrene (2.5 *μ*g/ml; Genomeditech). The stably transduced cells were selected using puromycin (1.5 *μ*g/ml; Genomeditech) over a 10 day period at 37°C. LM8-mock cells were untreated normal LM8 osteosarcoma cells.

### MTT assay

The effects of CCL2 on the proliferation of untreated LM8 cells (mock) or those transfected with LM8-NC, LM8-sh1 or LM8-sh3 were assessed using an MTT assay. The cells were seeded into 96-well plates at a density of 2,000 cells/well in DMEM-h and were cultured for 1, 2, 3, 4 or 5 days in DMEM supplemented with 10% FBS. Following incubation, 20 *μ*l MTT (5 mg/ml; Sigma-Aldrich) was added to each well and the cells were incubated for 4 h at 37°C. The culture medium was removed and 150 *μ*l dimethylsulfoxide (Sigma-Aldrich) was added, following which the optical densities (OD) were measured using an ELX800 Micro Plate Reader (Bio-Tec Instruments, Inc,. Winooski, VT, USA) at 490 nm. The cell viability was calculated using the following equation: Cell viability (%) = (OD_490nm_ treatment / OD_490nm_ control) × 100%.

### Spheroid forming assay

The cells were seeded into 6-well plates at a density of 300 cells/well. Following incubation for 15 days, the colonies were washed three times with phosphate-buffered saline (PBS; Sangon Biotech Co., Ltd., Shanghai, China), fixed with methanol/acetic acid (3:1) and stained with 1% crystal violet (Beyotime Institute of Biotechnology, Shanghai, China). The number of colonies (>10 cells) in four different field of views were quantified under a microscope (magnification, ×50; DP72; Olympus Corporation, Tokyo, Japan) and the mean value was calculated.

### Wound-healing assay

The cells (5×10^5^ cells/well) were seeded into 24-well plates and cultured until 90% confluent, at 37°C for ~24 h. The monolayer of cells was then scratched using a thin disposable tip to wound the layer, and the cells were incubated for 24 h at 37°C. Images of cell migration were captured using an Olympus microscope (Olympus, Tokyo, Japan). The migratory activity of the cells was expressed as the percentage of the area covered by cells, which was measured using Olympus DP72 software (Olympus).

### Transwell assay

The migration assay was performed using a Transwell (Corning Incorporated, Corning, NY, USA; pore size, 8 *μ*m) in 24-well plates. Prior to performing the migration assay, the cells were pretreated for 24 h in FBS-free medium, with or without 30 *μ*M AMD3100. The cells (~10×10^4^ cells in 200 *μ*l FBS-free DMEM-h) were placed in the upper chamber, and DMEM-h supplemented with 10% FBS was placed in the lower chamber. The plates were incubated for 48 h at 37°C in 5% CO_2_, following which the culture medium was removed and the filters were washed with PBS twice. The cells on the upper side of filters were removed using cotton-tipped swabs, and the cells on the underside of the filters were fixed in 95% alcohol for 10 min and stained with 0.1% crystal violet for 15 min at room temperature. The cells, which had migrated from the upper to the lower side of the filter were counted under a light microscope by counting 10 random fields of view at magnification ×100. The tumor cell migration assay was performed in triplicate.

### RNA isolation and reverse transcription-quantitative polymerase chain reaction (RT-qPCR)

The total RNA from the osteosarcoma LM8, DUNN, K7, K12 and K7M3 cell lines was isolated using TRIzol reagent (Invitrogen Life Technologies), according to the manufacturer’s instructions. Aliquots (2 *μ*g) of total RNA were reverse transcribed to cDNA using Superscript™ II Reverse Transcriptase (Invitrogen Life Technologies.). The expression of CCL2 was determined in 10 ng cDNA by qPCR using Brilliant RII SYBR Green qPCR master mix (Stratagene Corp., La Jolla, CA, USA) on an Applied Biosystems 7500 real time PCR system (Applied Biosystems, Foster City, CA, USA). The PCR cycling conditions were as follows: 95°C for 30 sec, 40 cycles of 95°C for 5 sec and 60°C for 30 sec. The GAPDH housekeeping gene was used as an internal control. The primer sequences were as follow: mouse CCL2 sense, 5′-AAA GAT GGG CTC CTC CTG TCC TG-3′, antisense, 5′-GTG CTG GGA TGG GAA GGT GGC TC-3′; and mouse GAPDH sense, 5′-CCT TCA TTG ACC TTC ACT ACA TGG TCT A-3′, and antisense, 5′-GCT GTA GCC AAA TTC ATT GTC GTT ACC A-3′ (Sangon Biotech Co., Ltd.).

### Western blot analysis

Western blot analysis was performed to detect the expression levels of CCL2, AKT and p-AKT in the osteosarcoma cell lines transfected with mock, NC, Lm8-sh1 or LM8-sh3. The cell lysates were obtained using radioimmunoprecipitation lysis buffer (Beyotime Institute of Biotechnology), supplemented with protease inhibitor and phosphatase inhibitor (Thermo Fisher Scientific). The protein concentrations were calculated using a Bicinchoninic Acid Protein Assay kit (Thermo Fisher Scientific). Equal quantities of total protein (30 *μ*g) from each sample were separated by electrophoresis on 10% sodium dodecyl sulphate-polyacrylamide gels (Sangon Biotech Co., Ltd.) at 80v and transferred onto nitrocellulose membranes (Shanghai Your Sun Biological Technology Co., Ltd., Shanghai, China). Following blocking with 5% non-fat milk in Tris-buffered saline with Tween-20 (1:1,000) for 1 h, the membranes were incubated overnight at 4°C with a 1:1,000 dilution of rabbit primary antibodies against CCL2, p-AKT, AKT and β-actin. The membranes were subsequently washed three times for 10 min each time with Tween 20-PBS (1:1,000), and then incubated with secondary goat anti-rabbit immunoglobulin (Ig)G antibody at room temperature for 1 h. The membranes were then washed with Tween 20-PBS three times for 10 min. The expression levels of the proteins were analyzed using an Odyssey infrared laser imaging system (Li-Cor Biosciences, Lincoln, NE, USA).

### Animal experiments

The use of mice (aged 4–5 weeks) were approved by the Ethics Committee of Shanghai Tenth People’s Hospital (Shanghai, China). A total of 10 female mice (average weight, 15 g) were housed in a standard animal laboratory with *ad libitum* access to food and water. The mice were maintained under constant environmental conditions with a 12 h light/dark cycle. All injections were performed under aseptic conditions by intraperitoneal injection of 4% chloral hydrate at 0.01 ml/g dose. MDA-MB-231 cells (1×10^6^), stably expressing the miR-374b or EV-control vector, were injected into the dorsal flank of nude mice. Each group contained five mice and the experiment was repeated in triplicate. The mice were sacrificed by cervical dislocation under anesthesia (intraperitoneal injection of 4% 0.01 ml/g chloral hydrate) 20 days later and the tumors were removed and weighed (PRACTUM124-1CN; Sartorius AG, Goettingen, Germany). The tumor size was measured every 3 days and the formula, volume = (Dxd2) / 2, was used to evaluate the tumor volume, where ‘D’ is the longest diameter and ‘d’ is the shortest diameter. Specimens of the lung in the xenograft tumor were fixed by formalin for 24 h, and then dehydrated by 70, 80 and 90% ethanol for 3 h respectively, and then 100% ethanol for 2 h two times. Following vitrification by xylene twice for 20 min each, and immersion in paraffin for 40 min twice, the specimens were embedded and sliced. Staining was performed as follows: Hematoxylin staining for 10 min, hydrochloric acid alcohol solution for 40 sec decoloring, eosin staining for 10 min and 90% ethanol for 40 sec decoloring (all Sangon Biotech Co., Ltd.). Subsequently, neutral balsam was used for mounting and the section was observed and photographed under the microscope. All animal experiments were performed according to the Animal Experimental Ethics Committee of Tongji University (Tongji, China).

### Statistical analysis

Statistical analyses were performed using SPSS 15.0 software (SPSS Inc., Chicago, IL, USA). The data were analyzed using an unpaired two-tailed Student’s t-test and data are expressed as the mean ± standard deviation. P<0.05 was considered to indicate a statistically significant difference.

## Results

### Expression of CCL2 in the osteosarcoma cell lines

**Figure 1:**
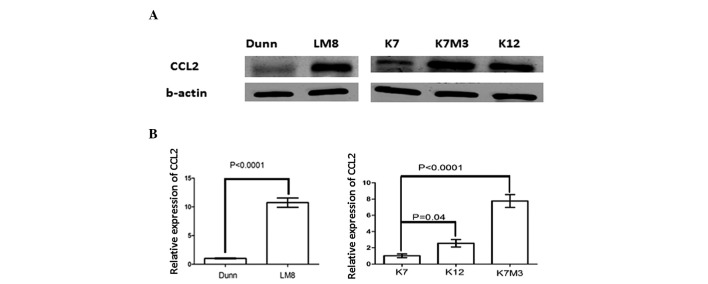
Detection of CCL2 in the osteosarcoma cell lines by western blot analysis. (A) Expression of CCL2 was downregulated in the DUNN and K7 osteosarcoma cell lines compared with the LM8, K12 and K7M3 cell lines (B) Reverse transcription-quantitative polymerase chain reaction demonstrated the same results (P<0.0001 LM8, K12 and K7M3, vs. Dunn and K7). Error bars represent the mean ± standard deviation. CCL, monocyte chemotactic protein.

Since the importance of chemokines in the occurrence and development of various types of cancer is increasing, it is important to investigate the role of CCL2 in the biology and pathophysiology of osteosarcoma. The present study performed RT-qPCR and western blot analysis to analyze the expression of CCL2 in the osteosarcoma cell lines. It was observed that the expression of CCL2 was higher in the more malignant osteosarcoma cell lines (LM8 and K7M3; Fig. 1A and B) compared with the less malignant cell lines (Dunn, K7 and K12).

### Knockdown of CCL2 reduces the proliferation of LM8 cells in vitro

**Figure 2:**
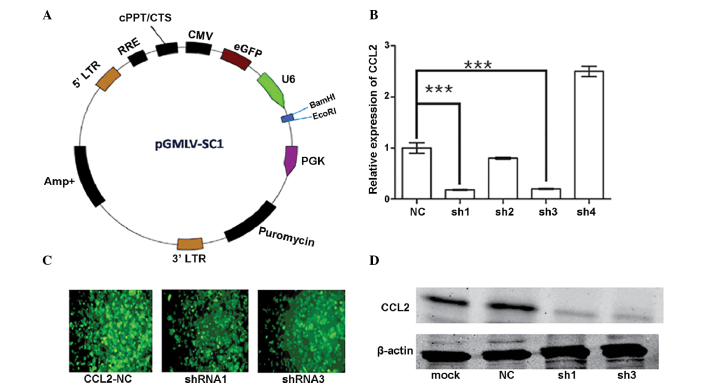
Detection of CCL2 in the LM8, LM8-NC, LM8-sh1 and LM8-sh3 cells. (A) Structure of the pGMLV-SC1 RNAi vector. (B) Relative expression of CCL2 detected in the LM8-NC, sh1, sh2, sh3 and sh4 cells. Reverse transcription-quantitative polymerase chain reaction revealed that the expression of CCL2 was knocked down in the LM8-sh1 and LM8-sh3 cells. (C) Immunofluorescence images demonstrating a reduction in the expression of CCL2 in the cells following transfection. (D) Western blot analysis confirmed the same results. ^***^P<0.0001; error bars represent the mean ± standard deviation. NC, negative control; Mock; untransfected; sh, short hairpin; CCL, monocyte chemotactic protein.

**Figure 3:**
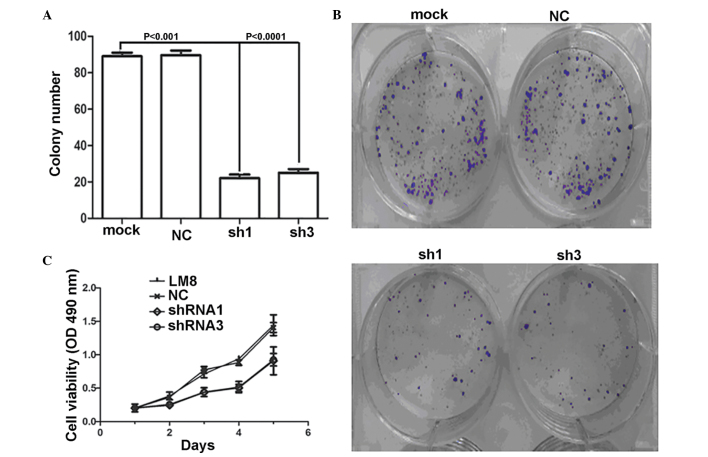
CCL2 promotes LM8 cell growth *in vitro*. (A and B) Colony formation assay of the LM8 cells transfected with negative control, CCL2-sh1 (P<0.01, compared with mock) or CCL2-sh3 vectors (p<0.0001, compared with mock). (C) Effect of CCL2 on the proliferation of LM8 cells was detected using an MTT assay. Error bars represent the mean ± standard deviation. NC, negative control; mock, untransfected; sh, short hairpin; OD. optical density.

Previous studies have demonstrated that chemokines and their receptors promote malignant tumor progression via promoting cell proliferation (14,15). The expression of CCL2 in the LM8, NC, LM8-sh1 and LM8-sh3 cells was detected by RT-qPCR and western blot analysis. As shown in Fig. 2B and C, the LM8 cells transfected with the plasmid encoding shRNA-CCL2 (pGMLV-SC1 CCL2) exhibited reduced mRNA and protein expression levels of CCL2 compared with the control cells transfected with a negative control plasmid (pGMLV-SC1 NC) and the untransfected LM8 cells. In addition, an MTT assay and a clonogenic survival assay revealed that the CCL2-knockdown cells exhibited a reduced cell proliferation rate compared with the mock and NC cells (Fig. 3A–C).

### Knockdown of CCL2 reduces the invasion of LM8 cells in vitro

**Figure 4:**
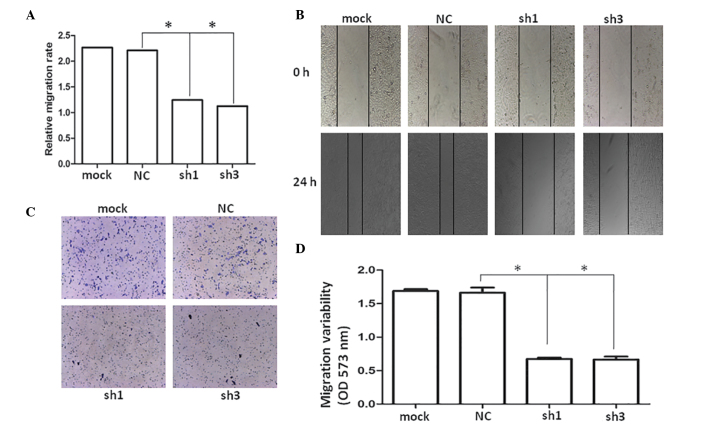
CCL2 promotes LM8 cells invasion *in vitro*. (A) Wound-healing assay results. (B) Five representative images of the scratched areas were captured under a light microscope at 0 and 24 h. The average gap of the time-points was used to quantify the wound healing ability of each group (magnification, ×50). (C) Transwell assay of the LM8, NC, LM8-sh1 and LM8-sh3 cells (magnification, ×100; crystal violet stain). (D) Quantification of migration variability. *P<0.05, compared with NC. mock, untransfected; NC, negative control; sh, short hairpin.

The highly invasive LM8 osteosarcoma cell line exhibit significant migratory ability, and CCL2 has been found to induce the migration of bladder and prostate cancer cells (16,17). Therefore, it was hypothesized that CCL2 may be involved in LM8 cell migration. As shown in Fig. 4B and C, the wound healing and transwell assays revealed that the CCL2-knockdown cells exhibited a significantly slower rate of migration compared with the mock and NC cells (Fig. 4A and D).

### CCL2 promotes the growth of osteosarcoma tumors in mice

**Figure 5:**
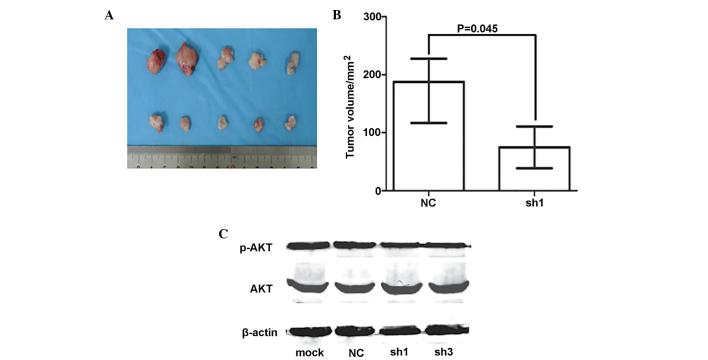
CCL2 promotes LM8 cell invasion *in vivo*. (A) Tumors were dissected from the mice and measured. (B) Tumor volumes were measured after 20 days. (C) Western blot analysis of the protein expression levels of AKT and p-AKT were detected in the LM8-mock, NC, LM8-sh1 and LM8-sh3 cells. Error bars represent the mean ± standard deviation. NC, negative control; mock, untransfected; sh, short hairpin; p-, phosphorylated.

The cells from each of the treatment groups were implanted into mice to investigate the extent to which CCL2 affects the growth or invasion of cell-derived osteosarcoma tumors. The results indicated that CCL2-shRNA LM8 cells formed smaller nodes compared with the CCL2-NC and mock LM8 cells (Fig. 5A and B). The necrotic degree of lung metastasis was detected by hematoxylin-eosin staining. Microtumors were not observed in the lungs of the of mice models and they may have been associated with the injection of tumor cells into the muscle.

### Expression of p-AKT is downregulated in the CCL2-shRNA LM8 cells

The significance of the AKT pathway in cancer cell proliferation and metastasis has been evaluated in several studies (18,19). The expression of p-AKT was detected in the CCL2-shRNA, CCL2-NC and mock LM8 cells by western blot analysis, which revealed that the expression of p-AKT was downregulated in the CCL2-shRNA LM8 cells (Fig. 5C) compared with the mock and NC cells.

## Discussion

Osteosarcoma is the most common malignant bone tumor and exhibits a high propensity for metastatic spread, predominantly to the lung and bones (20). As a CCL2 receptor, the expression of CCR2 was detected in 100% of the 27 osteosarcoma samples (21). The expression of CCL2 correlated with a poor prognosis and metastatic disease in patients with several types of cancer, including breast and prostate cancer (22,23). CCL2 is involved in other cancer types, including bladder and prostate, by promoting the migration and invasion of cancer cells (16,17). Previous studies demonstrated that CCL2 is synthsized by breast tumors and the stroma recruitment metastasis-associated macrophages, and this promoted metastasis *in vivo* and shortened the survival rate of tumor-bearing mice (13).

The role of CCL2 in osteaosarcoma cells remains to be elucidated. The present study demonstrated that CCL2 promoted the proliferation and invasion of osteosarcoma cells. The mRNA and protein expression levels of CCL2 were upregulated in high-grade osteosarcoma cells. It was also suggested that CCL2 may be important in osteosarcoma cells. A pGMLV-SC1 RNAi vector, which inhibited the expression of CCL2 was transfected into LM8 cells to investigate the importance of CCL2 *in vivo* and *in vitro*. It was demonstrated that inhibition of the expression of CCL2 inhibited LM8 cell proliferation, decreased clonogenic formation and invasion *in vitro*. In murine models, inhibition of CCL2 decreased the weight of the cancer nodes. To further examine the molecular mechanism underlying growth promotion by CCL2, the protein expression of p-AKT was demonstrated to decrease in LM8 cells with CCL2-inhibition. The activation of AKT signaling is a common hallmark in various human cancers, including osteosarcoma (24,25). In high-grade osteosarcoma cells, the inhibition of AKT signaling may be a potential target for cancer therapy (26).

Previous studies have demonstrated that Carlumab (CNTO 888), which is a human IgG_1_κ monoclonal antibody with high affinity and specificity for human CCL2, does not inhibit the CCL2/CCR2 axis or demonstrate antitumor activity as a single agent in metastatic castration-resistant prostate cancer (27). However, whether carlumab is enriched in the tumor microenvironment remains to be elucidated. In conclusion, the present study provided a novel insight into how CCL2 affected tumor progression. Whether activation of CCL2 affected the expression of AKT will be determined in a future investigation.
